# Epithelial–Mesenchymal Transition Mechanisms in Chronic Airway Diseases: A Common Process to Target?

**DOI:** 10.3390/ijms241512412

**Published:** 2023-08-03

**Authors:** Angélique Mottais, Luca Riberi, Andrea Falco, Simone Soccal, Sophie Gohy, Virginia De Rose

**Affiliations:** 1 Pole of Pneumology, ENT, and Dermatology, Institute of Experimental and Clinical Research, Université Catholique de Louvain, 1200 Brussels, Belgium; angelique.mottais@uclouvain.be (A.M.); sophie.gohy@uclouvain.be (S.G.); 2 Postgraduate School in Respiratory Medicine, University of Torino, 10124 Torino, Italy; luca.riberi@unito.it (L.R.); andrea.falco@unito.it (A.F.); simone.soccal@unito.it (S.S.); 3 Department of Pneumology, Cliniques Universitaires Saint-Luc, 1200 Brussels, Belgium; 4 Cystic Fibrosis Reference Centre, Cliniques Universitaires Saint-Luc, 1200 Brussels, Belgium; 5 Department of Molecular Biotechnology and Health Sciences, University of Torino, 10126 Torino, Italy

**Keywords:** epithelial-to-mesenchymal transition, fibrosis, chronic airway diseases, remodelling

## Abstract

Epithelial-to-mesenchymal transition (EMT) is a reversible process, in which epithelial cells lose their epithelial traits and acquire a mesenchymal phenotype. This transformation has been described in different lung diseases, such as lung cancer, interstitial lung diseases, asthma, chronic obstructive pulmonary disease and other muco-obstructive lung diseases, such as cystic fibrosis and non-cystic fibrosis bronchiectasis. The exaggerated chronic inflammation typical of these pulmonary diseases can induce molecular reprogramming with subsequent self-sustaining aberrant and excessive profibrotic tissue repair. Over time this process leads to structural changes with progressive organ dysfunction and lung function impairment. Although having common signalling pathways, specific triggers and regulation mechanisms might be present in each disease. This review aims to describe the various mechanisms associated with fibrotic changes and airway remodelling involved in chronic airway diseases. Having better knowledge of the mechanisms underlying the EMT process may help us to identify specific targets and thus lead to the development of novel therapeutic strategies to prevent or limit the onset of irreversible structural changes.

## 1. Introduction

Epithelial-to-mesenchymal transition (EMT) is defined as a reversible cellular complex process, during which epithelial cells lose the classic epithelial markers, acquire a mesenchymal phenotype and are then able to produce extracellular matrix (ECM) [1]. This process, initially identified in embryonic development (type I), is also involved in tissue repair and fibrogenesis (type II) or tumour progression (type III) [2,3,4,5]. Several conditions, including chronic inflammation, can induce this molecular reprogramming, followed by the subsequent induction of self-perpetuating profibrotic, pro-remodelling and pro-neoplastic events [4]. EMT has been described in the pathophysiology of different lung diseases, such as lung cancer and its progression [6,7,8], interstitial lung diseases [9], asthma [10] and chronic obstructive pulmonary disease (COPD) [11]. More recently, it has also been described in other muco-obstructive lung diseases, such as cystic fibrosis (CF) [12] and non-cystic fibrosis bronchiectasis (NCFB).

Chronic obstructive airway diseases are characterised by different complex mechanisms underlying their airway changes and remodelling [13]. Although fibrosis does not play the key role observed in interstitial lung diseases [14], this process and its associated mechanisms, including EMT, appears to be crucial in pathological airway alterations leading to the remodelling, distortion and narrowing of the airways as well as the subsequent lung function impairment observed in these diseases.

The aim of this review is to focus on EMT molecular mechanisms and their associated airway remodelling involved in chronic airway diseases, including bronchial asthma and muco-obstructive lung diseases, such as CF, NCFB and COPD. We believe in fact that this is a quite neglected area of research and tried to highlight the fields on which future research should focus. The following MeSH terms were used for this review in the selected databases (PubMed and ResearchGate): epithelial mesenchymal transition; asthma; chronic obstructive pulmonary disease; cystic fibrosis; non-cystic fibrosis bronchiectasis; airway remodelling; airway fibrosis and airway diseases. We have excluded studies published in a language other than French, Italian or English. We have also excluded studies talking about cancer-associated EMT in lung disease.

## 2. Common Genetic and Biochemical Mechanisms of EMT

Three different types of EMT have been described depending on the biological or pathophysiological context [15,16]. Type I EMT is involved in embryogenesis and participates in organogenesis, such as the formation of the mesoderm during gastrulation and the delamination of the neural crest [17,18]. During embryogenesis, epithelial cells engage in several successive cycles of EMT and mesenchymal–epithelial transition. Type III EMT is associated with cancer progression [19]. Finally, type II EMT, which we will discuss in this review, is important for tissue regeneration and organ fibrosis. When exposed to pathogens or a toxic agent, the epithelium is damaged, and it will seek to rebuild itself. To repair the wound, basal cells migrate, proliferate and finally differentiate to reconstruct a pseudostratified respiratory epithelium. Chronic aggression and/or excessive inflammation leads to fibrogenesis. Fibrosis is characterised by the proliferation and activation of fibroblasts and myofibroblasts as well as the production of an excessive and abnormal extracellular matrix (ECM) [20]. Myofibroblasts originating from specialised epithelial cell populations during EMT have profibrotic and pro-inflammatory activity; these cells are involved in production of ECM components, as well as in matrix remodelling through the production of proteins, such as MMPs and the tissue inhibitors of metalloproteinases [21]. Myofibroblasts can originate from multiple other sources besides epithelial–mesenchymal transition, including differentiation from local fibroblasts, the recruitment of fibrocytes from bone marrow, endothelial–mesenchymal transition or from macrophages (i.e., macrophage–mesenchymal transition (MMT)) as well as from pericytes that are also able to differentiate into myofibroblasts [22].

Genetic and biochemical elements are common in the generation of different types of EMT, a process that is characterised by molecular reprogramming involving the loss of expression of epithelial proteins (e.g., E-cadherin, claudin and occludin) and the activation of mesenchymal genes, such as α-smooth muscle actin (α-SMA), N-cadherin and vimentin, which further lead to the loss of intercellular junctions (e.g., adherence junctions, tight junctions and desmosomes) and thus of epithelial cell polarity [23]. This molecular reprogramming is regulated by the activation of different transcription factors (TFs), such as SNAIL factors, Zinc-finger E-box-binding (ZEB) factors and TWIST factors [23,24].

**Figure 1:**
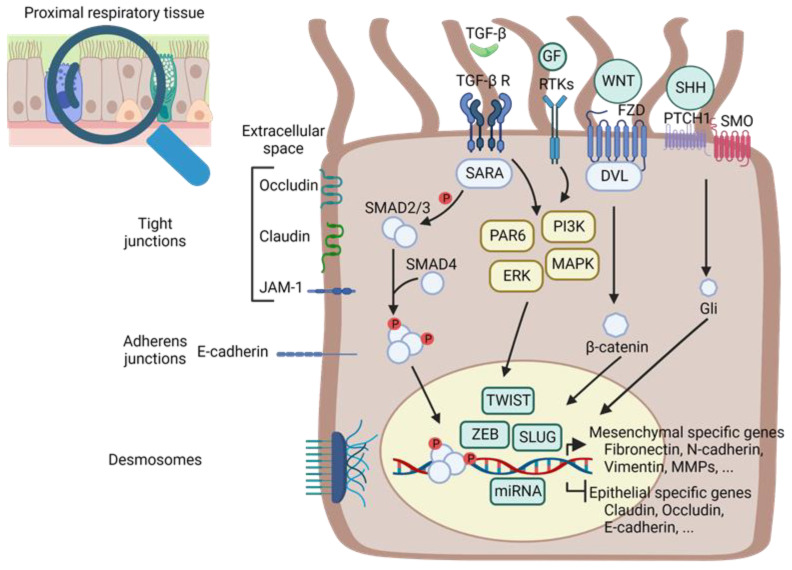
Signal pathways involved in type II EMT. TGF-β, other GF, WNT and SHH are particularly known as transcriptional-factor EMT inducers (e.g., SNAIL or ZEB, and miRNAs) that repress genes encoding epithelium-specific proteins, such as components of tight junctions (occludin, claudin and JAM-1), adhesion junctions and desmosomes, and activate mesenchyme-specific genes, such as N-cadherin and vimentin. These transcriptional changes lead to the reorganisation of the cytoskeleton and the production of ECM (created with BioRender.com and inspired by [7,11]). ERK, extracellular signal-regulated kinases; FZD, fizzled; GF, growth factor; JAM, junctional adhesion molecule; MAPK, mitogen-activated protein kinase; miRNA, microRNA; MMP, matrix metalloproteinase; PI3K, phosphoinositide 3-kinase; PTCH, Patched protein; RTK, receptor tyrosine kinase; SARA, SMAD anchor for receptor activation; SHH, Sonic Hedgehog; SMO, Smoothened protein; TGF, Transforming Growth Factor-β; WNT, wingless/integrated; ZEB, Zinc-finger E-box-binding.

The factors inducing EMT are multiple, and they are type- and organ-dependent. The pulmonary type II EMT results primarily from chronic lung inflammation, and it involves many pro-inflammatory actors (such as inflammatory cells, cytokines, chemokines and growth factors (GFs)) and different signal pathways, as summarised in Figure 1 [7,11].

The most widely described EMT-inducing factor is Transforming Growth Factor-β (TGF-β). The TGF-β family contains 33 TGF-β-related proteins, including 3 TGF-β isoforms (TGF-β1, 2 and 3). The proteins of the TGF-β family have multiple functions at the cellular and developmental level [25,26,27] as well as in the pathogenesis of diseases [28,29]. In the lung, TGF-β isoforms are involved in embryogenesis, including lung tissue development as well as in inflammation and tissue repair [30,31]. TGF-β1 is the first isoform to have been identified [25], and it has been found to play a crucial role in fibrotic lung diseases [26,32,33,34,35,36,37], particularly in airway remodelling [38,39]. Moreover, TGF-β1 is involved in the regulation of ECM components, as well as in fibroblast activation and in myofibroblast differentiation [40,41]. After synthesis, TGF-β dimerises via a disulphide bond and associates with the latency-associated peptide, which can attach to the latent TGF-β-binding protein [42]. These latent complexes are in a biologically inactive form that can be cleaved by various proteases to release active TGF-β [43]. The binding of TGF-β to its receptor (TGFBR1/2) can trigger two signalling pathways, the canonical SMAD-dependent pathway and the non-canonical pathways, which include phosphoinositide 3-kinase (PI3K/AKT) and mitogen-activated protein kinase (RAS/MAPK) (Figure 1). These two signal cascades lead to the repression of genes involved in the expression of epithelial phenotype and the activation of the expression of a mesenchymal phenotype.

Other signalling pathways activating TFs, such as the WNT/β-catenin [44,45] or Sonic Hedgehog (SHH) pathways may also be involved in the induction of EMT [46,47,48,49]. The binding of WNT to its receptor induces the release of β-catenin, which, once stabilised, migrates into the nucleus to regulate target genes. The binding of the SHH ligand to the Patched protein releases the Smoothened protein from the repressive action of the latter, resulting in the dissociation of the cytoplasmic complex, thus preventing the degradation of TFs that activate or repress the target genes.

Other GFs, such as the Platelet-derived Growth Factor (PDGF) [50], TGF-α [51,52,53], Fibroblasts Growth Factor-β (FGF-β) [54], Epidermal Growth Factor (EGF) [55,56,57] and Connective Tissue Growth Factor; and mediators, such as Transglutaminase-2 (TG2) [58,59,60] or reactive oxygen species (ROS) [61,62], participate in the EMT process and activate different pathways, such as JAK/STAT and PI3K/AKT, by binding to receptor tyrosine kinases. GFs also participate in the recruitment of neutrophils [63] and in the transformation of monocytes into macrophages. These cells produce cytokines (e.g., Tumour Necrosis Factor (TNF)-α, interleukin (IL)-1, IL-6 and GFs (e.g., TGF-β and PDGF)) that participate in the proliferation of fibroblasts and their differentiation into myofibroblasts [64].

## 3. Asthma

Asthma is a heterogeneous disease with a worldwide prevalence characterised by variable airflow obstruction, chronic airway inflammation and remodelling, and airway hyper-responsiveness [65].

Despite an increasing research interest in mechanisms of this disease and improved knowledge in this field, many questions still remain open. In particular, the processes leading to structural changes and airway remodelling are still not fully defined. The term airway remodelling summarises a wide variety of structural changes in both large and small airways of asthmatic patients; these include epithelium disruption [66], thickening of the reticular basement membrane (RBM) associated with subepithelial fibrosis, goblet metaplasia, angiogenesis, increased airway smooth muscle (ASM) mass [67,68,69] and ECM deposition [70]. Airway remodelling is progressively becoming a relevant research topic as it is present in all asthma phenotypes independently of disease severity, and it is also linked to progressive loss of lung function, airway hyper-responsiveness and the greater need for medications [71,72,73]. Furthermore, although airway remodelling has been considered a progressive consequence of chronic inflammation [74,75,76], more recent data have shown that features of remodelling are already observed in asthmatic children who are 2–4 years old, even in the absence of atopic inflammation or before clinical manifestations of the disease [77,78,79]. Broekema et al. studied a cohort of adult asthmatic patients followed up for 3 years and documented that the extent of structural changes due to airway remodelling remained unchanged independently of symptoms control or medication use [80].

EMT is believed to play an important contributory role to airway remodelling in asthma [10,81,82]; this process leads to the migration of increased numbers of mesenchymal cells into the subepithelial tissue and to enhanced production of ECM, contributing to airway wall fibrosis [83]. However, the potential drivers of EMT as well as its causal role in airway remodelling in asthma are a controversial issue, and some discrepancy exists in the available data.

### 3.1. Chronic Inflammation, EMT and Airway Remodelling

The chronic inflammatory process in response to persistent environmental triggers is a crucial driver of epithelial barrier injury and subsequent altered repair in asthma, leading to the loss of barrier function. EMT is one of the processes that have been suggested to contribute to the disruption of the epithelial barrier, however the intrinsic mechanisms of this process are not yet clearly defined. Furthermore, the evidence suggests that additional factors besides inflammatory insult may contribute to barrier dysfunction in asthma and trigger the EMT process (in vitro and in vivo evidence is summarised in Table 1). Loffredo et al. [84] used gene expression microarray datasets by several independent groups to gain insights into the processes involved in epithelial barrier dysfunction in asthma. Using this approach, they reported little evidence of classical EMT markers expression but identified a novel suite of potential biomarkers involved in epithelial–mesenchymal signal dysregulation, including Ephrin B2, FGF receptor 1, FGF receptor 2, Insulin Receptor, Insulin Receptor Substrate 2, NOTCH2, TLE family member 1 and neurotrophic receptor tyrosine kinase 2; this signature of asthma was present in mild to severe disease and seems to progress with disease severity. These findings thus suggest that factors others than EMT driven by chronic inflammation might be involved in epithelial barrier disruption in asthma. Following the chronic aggression by noxious external agents, the airway epithelium is able to modulate the inflammatory/immune responses by interacting with inflammatory/immune cells and by releasing several cytokines and GFs, such as TGF-β, EGF, FGF and PDGF [85,86], thus contributing to structural alterations of the airway walls and the remodelling process. Subepithelial fibrosis, with an increase in fibroblasts in the airway epithelium, myofibroblasts hyperplasia and ECM deposition are major pathological features of the remodelling process in bronchial asthma. As already discussed, EMT is a relevant mechanism in fibroblast and myofibroblast proliferation and activation.

TGF-β1, a key molecule driving this process, induces the typical changes in cells from asthmatic patients [87,88]. Increased TGF-β1 levels have been observed in bronchoalveolar lavage fluid (BALF) and bronchial biopsies from asthmatic patients [89]. Interestingly, the Th2 cytokine IL-4 and the Th17-derived IL-17A, which are crucial mediators of the inflammatory process in severe asthma, also represent the two major cytokines inducing EMT and airway remodelling through TGF-β1 expression [90,91]. TGF-β1 synergises with IL-4 and IL-17 in suppressing E-cadherin expression and inducing α-SMA, vimentin and fibronectin expression in epithelial cells [92]. This mediator has also been shown to synergise with house dust mite (HDM) extracts in enhancing the expression of EMT markers [93,94].

The role of the EMT process in airway remodelling and subepithelial fibrosis in asthma has been further confirmed by the study of Johnson et al. [95], that in a transgenic murine model sensitised to HDM showed that large airway epithelial cells progressively lost their typical features, acquiring the expression of mesenchymal markers, such as vimentin, α-SMA and type I pro-collagen. An increased expression and nuclear translocation of SNAIL1, a transcriptional factor that is a potent inducer of EMT, was also observed in the airway epithelial cells of HDM-exposed mice. Interestingly, fate-mapping studies documented that epithelial cells migrated into the subepithelial compartment of the airway wall [95].

As already highlighted, GFs, such as TGF-β1, are also involved in EMT induced by inflammatory cells, and it has been reported in several studies that TGF-β1 expression correlates with the number of eosinophils and the degree of airway remodelling in bronchial asthma [96,97]. To evaluate the effect of eosinophils on EMT, Yasukawa et al. [98] carried out in vitro and in vivo studies. In particular, they assessed EMT in mice instilled with bone-marrow-derived eosinophils and inhuman bronchial epithelial cells (BEC) co-cultured with eosinophils. The intratracheal instillation of eosinophils induced enhanced bronchial inflammation, EMT features and fibrosis associated with increased concentration of GFs; interestingly, the instillation of eosinophils pre-treated with TGF-β1 siRNA was associated with reduced airway wall fibrosis. When co-cultured with BECs, eosinophils induced EMT in cells that were associated with enhanced TGF-β1 expression and SMAD3 phosphorylation. These findings suggest that eosinophils are capable of inducing fibrotic changes and EMT in airway epithelial cells, thus contributing to airway remodelling in asthma.

In an in vitro study using BECs, IL-1β was able to induce a decreased expression of E-cadherin, associated with an increased expression of some ECM component such as tenascin C; these effects were enhanced when the cells were co-stimulated with both TGF-β and IL-1β, further supporting the concept that the inflammatory context is crucially involved in the EMT process in asthma [99]. However, it is worth highlighting that in the same study, glucocorticoids were not able to induce any effect on EMT. Thus, further studies are needed to define the role of inflammatory pathways resistant to steroids in EMT.

IL-24 is a pleiotropic cytokine, member of the IL-10 family, that has been implicated in the induction of tissue fibrosis and remodelling [100], and increased levels of this cytokine have been reported in nasal secretions and in the induced sputum of asthmatic patients [101]. Very recently, Feng and co-workers [102] showed that IL-24 was able to promote the expression of EMT mesenchymal markers in BEAS-2B cells via the STAT3 and extracellular signal-regulated kinases (ERK)1/2 pathways. Furthermore, in vivo, IL-24 was highly expressed in mouse airway epithelium in a HDM-induced model of asthma, and this was associated with an upregulation of EMT markers. Interestingly, IL-37 reduced the airway remodelling by inhibiting IL-24-mediated EMT. These findings thus suggest that IL-24 contributes to airway remodelling and may represent a novel therapeutic target for preventing and treating this process in asthma, suggesting, on the other hand, a potential therapeutic effect of IL-37 on airway remodelling [102].

ECM, a key component of airway remodelling, is involved in a crosstalk with airway epithelial cells. Changes in its composition in fatal asthma have been demonstrated in various studies [103,104,105,106,107,108,109]. As already emphasised, fibroblasts and myofibroblasts are the most important cells involved in the production of ECM and both play a crucial role in the process of airway remodelling. An increase in myofibroblasts has been documented in the conducting airways of asthmatic patients [110,111] and is mostly responsible for the increase in type I collagen, type III collagen and fibronectin observed at this level; these cells have been found close to the RBM and ASM cells [112,113].

In vivo and in vitro observations in different asthma models have also documented the recruitment of circulating fibrocytes into the airways of asthmatic patients. Nihlberg and co-workers [114] have demonstrated that in bronchial biopsies of patients with mild asthma, fibrocytes were localised close to the RBM, and their numbers correlated with RBM thickness. These cells were also present in the BALF of these patients, supporting a potential role of fibroblasts progenitors in the early stage of airway remodelling in asthma.

Changes in ECM in asthma are not limited to the increase in fibrillar collagen but are also characterised by an altered deposition [103]; a study using nonlinear optical microscopy [72] described a lack of production of decorin by fibroblasts. This molecule is involved in collagen formation, and its deficiency causes a more fragmented and disorganised collagen deposition in the *lamina propria* of asthmatic airways. This altered deposition, in turn [72], stimulates myofibroblasts formation and the subsequent ECM synthesis. This aberrant mechanism thus induces a vicious cycle of persistent deposition of disorganised collagen and airway remodelling leading to increased basal membrane thickening [72,115].

The alterations of the ECM and the thickening of the RBM seem to be correlated with airway hyper-responsiveness [116,117]. Interestingly, a great variance in RBM thickness has been observed among patients as well as in different areas of the airway walls in the same patient, suggesting that ECM alteration is a dynamic process that may proceed at different rates in different areas of the airway wall. No differences were observed between fatal and non-fatal asthma or according to patients’ age or gender [118]. Respiratory viruses, in particular *Rhinovirus*, contribute to the ECM remodelling in asthma, by increasing fibronectin, perlecan and type IV collagen deposition, as well as promoting ASM cell migration [119].

Another key component of airway remodelling—that is however less relevant in the context of this review—is the ASM [67,68,69]. In several studies carried out on bronchial biopsies, ASM mass correlates with asthma severity and airway hyper-responsiveness [120] and may increase from 5% up to 12% in patients with fatal asthma. This increase is mediated by both the hyperplasia and hypertrophy of ASM cells under the stimulus of various cytokines and GFs, including TGF-β1, EGF and PDGF [121,122,123,124]. ASM cells contribute to airway remodelling in asthma also through the modulation of the inflammatory process; in fact, these cells are able to release inflammatory cytokines such as IL-1β, TNF-α, IL-5, IL-13 and TGF-β [125,126,127]. Furthermore, it has been shown that repeated airway bronchoconstriction leads to a higher expression of TGF-β by epithelial cells, further enhancing subepithelial fibrosis and basal membrane thickening [128]. Bronchoconstriction may thus represent a trigger for airway remodelling independent of the inflammatory process [129].

Mucus hypersecretion is observed throughout the conducting airways and occurs at any stage of the disease from mild to fatal asthma [130,131,132,133,134]. It may be mediated by goblet cells hyperplasia and metaplasia, which is another feature of airway remodelling in asthma [68,135].

**Table 1:** Animal and cells culture models suggesting EMT implication in asthma.

Models	Techniques	EMT Program	AR	EMT Signal	EMT TFs	EMT Mark	Refs.	Year
HBECs of asthma and control subjects monolayer and ALI culture	RT-qPCR WB SDS-PAGE	+TGF-β1 10–50 ng/mL 72 h: spindle-shaped appearance 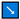 E-cadherin and ZO-1 (protein)						
	IHC and IF	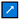 fibronectin, vimentin and α-SMA (mRNA and protein)	x	x		x	[87]	2009
		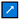 collagen-1α1, fibrinogen, connective tissue growth factor and TGF-β1 (mRNA) Smad3-dependent process						
16HBE 14o- cell line	RT-qPCR Cell proliferation WB ELISA	TGF-β1, IL-4 and IL-17A stimulation (72 h): proportion with a spindle-shape, fibroblast-like morphology with reduced cell–cell contact 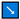 E-cadherin (mRNA and protein) 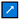 α-SMA (mRNA and protein) 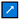 pERK1/2	x	x		x	[90]	2013
16HBE 14o- cell line	IF WB SDS-PAGE	TGF-β1 5 ng/mL: 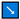 E-cadherin (protein) 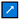 vimentin and fibronectin (protein) 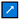 glycogen synthase kinase-3β (protein)	x	x		x	[93]	2010
ALI normal HBECs	WB	+TGF-β1 + HDM 50 μg/mL: 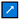 vimentin (protein) 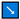 cytokeratin 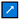 fibroblast-specific protein-1 (protein) delocalisation of E-cadherin	x	x		x	[93]	2010
Male and female transgenic mice stably expressing LacZ in lung epithelial cells (SPC-Cre; R26stop-LacZ) 8–12 weeks	IF	+25 µg/day HDM intranasally (10–15 weeks): inflammation epithelial damage and thickening of the sub-epithelial contractile smooth muscle layer tissue 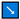 occludin and E-cadherin (protein) 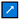 vimentin, α-SMA and pro-collagen I 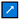 TGF-β1 in BALF Activation of Smad-dependent TGF-β signalling pathways (p-Smad3 and SNAIL1 proteins in the nuclei)	x			x	[95]	2011
Male C57/BL6 mice (9 weeks)	RT-qPCR WB ELISA	+bone-marrow-derived eosinophils (intratracheal instillation): inflammation in BALF 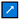 TGF-β1 in BALF type I collagen deposition 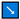 E-cadherin 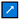 α-SMA and vimentin +TGF-β1 siRNA less airway remodelling	x	x		x	[98]	2013
BEAS-2B cell line	RT-qPCR WB ELISA	+primary human eosinophils or TGF-β1 or EoL-1 cells: fibroblast-like morphology and filamentous actin forming long stress fibres 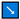 E-cadherin (mRNA and protein) 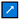 vimentin (mRNA and protein) Need for cell-to-cell contact for induction of EMT +PI3K and JNK inhibitors: EMT blocked	x	x		x	[98]	2013
BEAS-2B cell line	RT-qPCR WB IF Wound-healing assay	rhTGF-β1 5 ng/mL: spindle-fibroblast-like morphology with reduced cell–cell contact 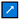 α-SMA (mRNA and protein) 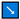 E-cadherin (mRNA and protein) 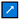 collagen type I, fibronectin-EDA and tenascin C (mRNA) 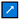 motility 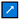 MMP-2/9 (protein) +IL-1β: 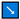 E-cadherin (mRNA 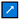 tenascin C expression (mRNA) Corticosteroid pretreatment does not abrogate TGFβ1-induced EMT	x	x		x	[99]	2009
Primary normal HBEC monolayer	RT-qPCR WB	rhTGF-β1 2 ng/mL: 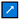 α-SMA (mRNA and protein) 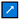 vimentin (mRNA) 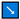 E-cadherin (mRNA and protein) 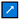 collagen type I, fibronectin-EDA and tenascin C (mRNA) 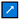 MMP-2/9 release (protein)		x		x	[99]	2009
BEAS-2B cell line	RT-qPCR IF WB Wound-healing cell migration assays	+IL-24 (dose–response): 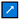 migratory capability Acquisition of a larger and more spindle-shaped morphology 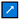 vimentin and α-SMA (mRNA and protein) 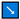 E-cadherin (mRNA and protein) Activation of STAT3 and ERK1/2 phosphorylation +IL-24 + inhibitors of JAK or ERK1/2: WT phenotype restored	x	x	x	x	[102]	2022
Female wild-type SPF BALB/c mice 6–8 weeks	IHC BALF Assessment of airway hyper-responsiveness	+25 μg/day HDM-induced asthma group 5 weeks: 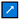 Airway resistance and inflammation 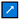 IL-24 protein Collagen deposition 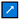 TGF-β1 (protein in BALF) 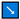 E-cadherin (protein) 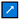 vimentin and α-SMA (protein) 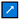 p-STAT3 and p-ERK1/2 (protein) +HDM + si-IL-24 or rhIL-37: 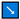 HDM-induced dysregulations	x	x	x	x	[102]	2022
Female C57BL/J mice 6–8 weeks CD146-KO C57BL/J mice IL-33 KO C57BL/J mice	WB IHC and IF ELISA	+25 μg intranasally administered HDM 5 weeks: 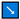 inflammation in both KO mice 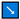 collagen I (protein) in both KO mice		x			[136]	2020
MLE-12 (mouse pulmonary epithelial cell line) and A549	WB IF RT-qPCR	+HDM 10–100 µg/mL: 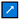 CD146 (mRNA and protein) 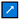 MyD88, phosphorylation of NF-κB p65 and p38 (protein) 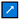 TGF-β and pSMAD3 (protein)						
Primary alveolar epithelial cells from mice		+IL-33 0.1–100 ng/mL: 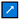 CD146 (protein in cells and secreted) +siRNA CD146: 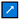 E-cadherin; expression inversely correlated with CD146		x			[136]	2020
Normal primary HBEC monolayer	RT-qPCR IF ELISA	+TGF-β1 10 ng/mL or neutrophils of asthma patients 48 h: 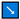 E-cadherin (mRNA) 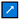 N-cadherin, α-SMA and vimentin (mRNA) morphological changes	x			x	[137]	2019
BALB/c mice 6–8 weeks	ELISA IHC RT-qPCR WB	+25 μg HDM intranasal instillation 4 weeks: 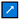 TF (mRNA and protein) +HDM + shRNA TF: 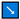 inflammation Improved hyperplasia collagen biomarkers of EMT reversed	x	x		x	[138]	2021
16HBE14o- cells	RT-qPCR Wound-healing and invasion assay	+TGF-β1 and HDM: 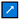 fibronectin 1, TF and TGF-β1 (mRNA and protein) shTF reverse the changes		x		x	[138]	2021
16HBE14o- cells	RT-qPCR WB	+TGF-β1: 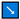 miR-448-5p (mRNA) 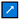 Six1 (mRNA and protein) +TGF-β1 + miR-448-5p: 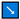 TGF-β1, pSMAD3 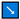 E-cadherin (mRNA) 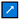 vimentin (mRNA) +Six1 silencing: similar phenotype to miR-448-5p overexpression		x		x	[139]	2019
BEAS-2B cell line	WB	LPS stimulation:						
	IHC	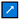 TGF-β1, TGF-β RI and TGF-β RII (protein) +10 ng/mL TGF-β: change of morphology PAR-1 induction 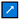 α-SMA (protein)	x	x		x	[140]	2014
Male BALB/c mice 6 weeks	IHC	+ovalbumin challenge: 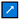 TGF-β1, TGF-β RI Epithelial thickening, collagen IV deposition 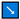 E-cadherin (protein) 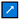 α-SMA (protein) PAR-1 induction	x	x		x	[140]	2014

α-SMA, Alpha Smooth Muscle Actin; ALI, air–liquid interface culture; AR, airway remodelling; BALF, bronchoalveolar lavage fluid; ELISA, enzyme-linked immunosorbent assay; EMT, epithelial–mesenchymal transition; HBECs, human bronchial epithelial cells; HDM, house dust mite extract; IF, immunofluorescence staining; IHC, immunohistochemistry; IL, interleukin; JNK, c-Jun N-terminal kinases; KO, knock-out; Mark, EMT-markers; MMP, matrix metalloproteinase; pERK1/2, phospho extracellular signal-regulated kinase; PI3, phosphatidylinositol3; Rh, recombinant human; RT-qPCR, quantitative real-time-PCR; SDS-PAGE, sodium dodecyl sulphate polyacrylamide gel electrophores; Signals, EMT-inducing signals; STAT3, signal transducer and activator of transcription; TF, tissue factor; TFs, EMT-transcription factors; TGF, transforming growth factor; WB, Western blot; WT, wild-type; ZO, zonula occludens.

### 3.2. Potential Effect of Pharmacological Treatment on EMT and Airway Remodelling

Relevant progresses have been obtained in asthma treatment during the last decades; at present, this treatment is focused on chronic inflammation and bronchial hyperresponsiveness and aims to achieve the best possible disease control and to minimise exacerbation risk and the development of persistent airflow limitation, whereas unfortunately, no drugs are yet available that primarily target airway remodelling.

The cornerstone of pharmacological treatment of asthma is represented by inhaled corticosteroids (ICS) that decrease airway inflammation inducing disease control. Several studies evaluated the potential effects of ICS on airway remodelling, showing a decrease in RBM thickness [116] and type III collagen deposition [141] in some studies that, however, were not confirmed in others [107,142,143]. ICS treatment has also been shown to partially restore the epithelial damage through the inhibition of inflammatory responses [144]. Despite these potential effects, longitudinal studies have demonstrated that lung function impairment persists over time if present in childhood despite treatment with ICS and bronchodilators [145,146]. Therefore, it seems that ICS do not have a definite impact on airway remodelling.

Similarly, data on the effects on airway remodelling of other drugs used in asthma treatment, such as antileukotrienes and macrolides, are scarce and have been mostly obtained in murine models. Antileukotrienes have been documented to decrease ASM mass in the large airways and to induce a reduction in RBM thickness in animal models of asthma [147]. A decrease in goblet cells hyperplasia and collagen deposition mediated by TGF-β inhibition was also observed with these drugs [148]. Azithromycin seems to reduce ASM cells viability and proliferation as well as ASM thickness in both proximal and distal airways in murine models of asthma [149,150]. A deficit in vitamin D seems to correlate with worsening of severe asthma, and in vitro studies have shown that this compound slowed down airway remodelling by inhibiting Nuclear Factor-κB activation and decreasing ASM cells proliferation as well as the secretion of some inflammatory mediators; thus it has been suggested that it could represent a potential treatment for airway remodelling in asthma [151,152,153]; however, further studies are needed to confirm these findings.

In the last 20 years, biological therapies have become available for specific endotypes of severe asthma, with relevant impact on clinical outcome. However, only a few studies have evaluated their effects on structural changes and airway remodelling. The first drug available in clinical practice was omalizumab, an anti-IgE antibody. In vitro studies have demonstrated that this drug may prevent ASM cells proliferation and the deposition of type I collagen and fibronectin in response to IgE [154]. Other studies, using high-resolution computed tomography (HRCT), showed a reduction in airway wall thickness and an increase in airway luminal area in patients treated with this drug [155,156,157]. Haldar et al. evaluated the effects of the anti-IL-5 antibody mepolizumab by using HRCT and detected an improvement in total airway surface in treated patients [158]. Using a bronchial biopsy, Flood-Page et al. documented a significant reduction in tenascin, lumican and type III pro-collagen expression in mild asthmatic patients as well as a decrease in TGF-β1 mRNA in eosinophils and in TGF-β1 levels in BALF [159]. Chachi et al. studied the effects of benralizumab, an anti-IL-5R antibody, on bronchial biopsies, showing an increase in ASM cells apoptosis with a consequent ASM mass reduction and suggested that this effect was likely the consequence of an indirect effect on eosinophilic inflammation [160].

Thus, the current knowledge is limited, and further studies are needed to clearly define the effects of current asthma treatment on airway remodelling. The development of new drugs primarily targeting the structural changes in the airways is a very important goal of future asthma pharmacological therapies and may represent a revolutionary approach to the treatment of this disease.

## 4. COPD

COPD is a leading cause of morbidity and mortality worldwide, and it is associated with an increasing social and economic burden [161]. The most important risk factor for COPD development is cigarette smoke, although other factors are also important, such as indoor and outdoor air pollution, and occupational exposure [161,162]. The persistent exposure to potential noxious agents in patients with COPD, associated with an individual susceptibility, induces tissue injury and an aberrant repair that cause both airway and lung parenchymal damage in different combinations and lead to chronic respiratory symptoms and the progressive impairment of lung function. Although pathological alterations occur throughout the respiratory tract in COPD, it is well known that the major site of injury and obstruction are the small airways, with peribronchiolar fibrosis and airway narrowing and obliteration [163,164]. Large airways are also affected in COPD, and characterized bysquamous metaplasia, mucus hypersecretion and ASM hyperplasia [163,164].

### 4.1. Chronic Inflammation in COPD

A chronic inflammatory process occurs both in the airways and the lung parenchyma in COPD as a consequence of persistent exposure to cigarette smoke and other noxious agents. A complex interplay of different inflammatory cells and mediators leads to small airway disease and emphysema, contributing to the progressive deterioration of lung function. Neutrophils are the main inflammatory cells present in the airway lumen and infiltrating the bronchial epithelium, glands and ASM; these cells largely mediate the structural damage through the release of several mediators, particularly proteolytic enzymes and ROS. Macrophages and T and B lymphocytes are also increased in small airways of patients with COPD and contribute to the enhancement of the inflammatory process as well as to the structural changes [163,164]. Macrophages are important effector cells in COPD; they produce several chemokines and cytokines, express high levels of mRNA for MMPs and release proteolytic enzymes and ROS, thus increasing tissue damage. These cells also release FGFs and other mediators involved in the proliferation of fibroblasts and their conversion to myofibroblasts and are thus major drivers of fibrosis in COPD. Moreover, macrophages contribute to the recruitment of other immune cells to the site of injury, thus enhancing the inflammatory process [22,165]. Lymphocytes and dendritic cells are organised into lymphoid follicles, which increase in severe disease, suggesting the relevance of the adaptive immune response in COPD. Furthermore, B cell infiltration in the walls of terminal bronchioles and alveoli is observed, which correlates with a loss of alveolar attachment to the airway walls. Increased numbers of type 1 and type 17 helper T cells have also been reported in patients with more severe disease [166]. Another cell type that may play a role in tissue alterations in COPD are mast cells, which have been implicated in airway vascular remodelling in this disease [163]. Mast cells secrete several bioactive molecules, including VEGF, FGF-β, TGF-β and also anti-angiogenetic factors [165,167]. Soltani and colleagues have documented that mast cell density in the *lamina propria,* and the RBM was greater in COPD patients compared to that of the controls and was related to the hypervascularity observed in the RBM, suggesting that perivascular mast cells may be involved in increased angiogenesis [168].

### 4.2. EMT and Fibrosis in COPD

As previously outlined, the pathogenesis of COPD is the result of complex and heterogeneous mechanisms that can interact each other; varying degrees of inflammation, tissue injury and abnormal repair with remodelling and fibrosis occur that involve the airways, lung parenchyma and lung vasculature. EMT has been suggested as an important mechanism contributing to the airway remodelling and fibrotic changes in COPD (recapitulated in Table 2 and Table 3). Several noxious agents, in particular cigarette smoking, can induce typical features, such as altered epithelial barrier function, the acquisition of mesenchymal phenotype by epithelial cells and an increased production of ECM, and there is evidence to support that EMT is activated in the airway tissue of COPD patients (recently reviewed in [11]).

As we already mentioned, small airways are the site of major involvement in COPD; remodelling with peribronchiolar fibrosis at this level is related to bronchiolar distortion and airway obstruction. The mechanisms of small airways fibrosis are not completely defined and several factors may play a role; recent studies, however, suggest that EMT is involved in these structural changes. The expression of EMT-related TF SNAIL1 and TWIST is upregulated in smokers with and without COPD and has been found to be associated with EMT activity and the levels of airflow obstruction [169]. Interestingly, the expression of SNAIL1 was higher in COPD patients with α1-antitrypsin deficiency than in patients without the deficiency [170]. E-cadherin, a prominent hallmark of EMT, was found to be decreased in COPD airways in several studies [171,172]. Shirahata et al. showed that also plasmatic sE-cadherin levels were lower in COPD patients and were related to the severity of airflow limitation [173].

BECs from COPD patients showed upregulated mesenchymal markers, such as α-SMA, type I collagen, vimentin, and NADPH Oxidase 4 as well as a lower expression of epithelial markers, confirming an active EMT in these patients [174]. Mesenchymal markers, such as S100A4, vimentin and α-SMA proteins as well as ECM proteins were also found increased in vivo in smokers with normal lung function and COPD patients (Table 3).

A major feature of EMT activity in vivo is represented by RBM fragmentation, associated with mesenchymal markers expression [16]. Sohal et al. showed that these pathological features are present in endobronchial biopsies from smokers with normal lung function and were even more evident in smokers with COPD [175]. Interestingly, membrane fragmentation in COPD has been shown to correlate with smoking history [176]. In a subsequent study, these authors demonstrated that in the RBM of smokers with COPD, there are cells that double stain for both mesenchymal and epithelial markers, further supporting the evidence of an active EMT in smoking-related COPD [177]. Cigarette smoking is not only capable of activating several signalling pathways involved in EMT per se, but it is also associated with a high oxidative burden and a chronic inflammatory process into the COPD airways that significantly contribute to the EMT process and the structural changes in the airways and lung parenchyma. One of the most important pathways affected by cigarette smoking is represented by the TGF-β/SMAD signalling pathway. As we already emphasised, TGF-β, and in particular TGF-β1, plays a crucial role in fibrotic diseases, and it is considered a key regulator of airway remodelling in COPD [171]. TGF-β is involved in the regulation of ECM components, as well as in the fibroblasts activation and in myofibroblasts differentiation [178,179]. It has been shown that TGF-β1 is capable of inducing EMT in cultures of human bronchial and lung epithelial cells in vitro [11]. Interestingly, cigarette smoking similarly induces EMT in lung and BEC through the TGF-β1/SMAD signalling pathway both in vitro and in vivo [11]. Furthermore, Takizawa et al. documented that mRNA levels of TGF-β1 were significantly higher in small airway epithelium from smokers with and without COPD than in non-smokers and were related to the degree of small airway obstruction, suggesting a tight relationship between smoking and TGF-β1 expression in small airways [180]. As previously mentioned, TGF-β1 also induces the differentiation of fibroblasts into myofibroblasts, motile and contractile cells that, once activated, secrete excessive and altered ECM (reviewed in [31]). As already discussed, these cells, that are crucial actors in the fibrotic process into the airways, can also originate from resident macrophages; during lung injury, in fact, macrophages may acquire a pro-inflammatory phenotype that promotes the differentiation and activation of these cells [181]. The process of MMT, which involves the transformation of macrophages into myofibroblasts, has recently been described in the lung and may contribute to development of fibrotic changes [182,183]. Although the molecular mechanisms involved in MMT are still unknown, this new evidence suggests novel potential targets for the development of antifibrotic therapies. Several studies suggest that the contribution of myofibroblasts and the EMT process may be important in both small and large airways fibrotic changes in COPD [175,177,184,185].

Recently, Eapen and colleagues [186] documented an increase in α-SMA+ myofibroblasts in the small airways of patients with COPD that was associated with the increased deposition of ECM proteins, EMT activity in epithelial cells and thickening of the *lamina propria*. These changes were also related to lung function impairment, further supporting an important role of EMT in small airways remodelling and narrowing.

Fibroblasts play also a key role in the fibrotic process; interestingly, Togo et al. demonstrated that lung fibroblasts of COPD patients have impaired repair mechanisms and suggested that this defect could contribute to the development of emphysema [187]. However, it is not known whether small airways fibroblasts are also defective in repair mechanisms, and it is not yet known whether these cells differ from interstitial lung fibroblasts. In this context, it is worth mentioning the observation of Lynch and colleagues who emphasised the possibility of fibroblast heterogeneity even within the same tissue [188,189].

As we already mentioned, cigarette smoke induces a high oxidative burden into the airways of COPD patients that is further enhanced by the persistent recruitment of inflammatory cells [190]. The oxidative stress drives additional inflammatory mechanisms in COPD; induces the expression of senescence markers in small airway fibroblasts; promotes profibrotic markers, including TGF-β and COL3A1, and is also associated with an impairment of antioxidant defences superoxide dismutase 2 and 3. These findings suggest that oxidative stress may contribute to small airway fibrosis in COPD [188] and contributes to the promotion of EMT [179]. Furthermore, the interaction between the oxidative stress and TGF-β is crucial in promoting fibrosis, inducing a self-perpetuating process by which TGF-β favours the production of ROS with increased oxidative stress that, in turn, activates latent TGF-β (reviewed in [31,191]). TGF-β can also induce EMT via non canonical pathways, such as the ERK, p38 MAPK, PI3K, Notch and WNT signalling pathways [192]. Recently, several studies suggested that the WNT/β-catenin pathway is activated in smokers and COPD patients and appears to be related to the EMT activity and the airway obstruction. The expression of the genes and proteins involved in this pathway is increased in the airway epithelium of smokers with COPD [193]. Interestingly, in vitro studies demonstrated that cigarette smoking and nicotine were able to induce EMT in human BEC by activating the WNT-3A/β-catenin pathway [194]. On the other hand, in peripheral tissue, a decrease in WNT/β-catenin signalling was observed that was associated with parenchymal alterations and the disruption of repair mechanisms, leading to an increase of emphysema in COPD patients [195].

PI3K/AKT is a signalling pathway involved in the regulation of several biological functions, including EMT. Also, in this case, some evidence suggests that cigarette smoke induces EMT through this pathway in COPD (Table 2 and Table 3). Milara and colleagues assessed in vitro the CSE-induced EMT in primary human BECs from small bronchi and suggested that the CSE effect is partially mediated by the activation of the PI3K/AKT/β-catenin pathway and the generation of ROS [196]. Recent studies added novel evidence on the potential mediators involved in EMT induction and airway remodelling in COPD: Jiang and co-workers [197] investigated the potential role of cathelicidin in inducing EMT in COPD. This protein is involved in various biological functions, including the regulation of inflammation and immunity and the promotion of tissue repair, and its overexpression in the airway epithelium has been implicated in mucus hypersecretion and fibroblast collagen production in smoking-related COPD [198,199]. Jiang and colleagues evaluated the expression of cathelicidin and EMT markers in human lung tissues from smokers with and without COPD, and in a COPD mouse model. They showed an upregulation of cathelicidin expression associated with EMT markers in the small airways of smokers with and without COPD. Significant smoking-induced EMT was also observed in the airways of mice. Interestingly, EMT was inhibited by the downregulation of CRAMP (the murine homologue of cathelicidin) in COPD mice. Finally, the authors demonstrated that cathelicidin promoted EMT by activating Tumour necrosis factor alpha (TNF-α) converting enzyme (TACE), Transforming growth factor alpha (TGF-α), and Epidermal growth factor receptor (EGFR) signalling pathways. Chu and colleagues evaluated the effect of CSE and IL-17A on bronchial EMT in a mice model of COPD and showed an increased expression of IL-17A in lung tissues and a synergistic effect of this cytokine and CSE on the induction of bronchial EMT [200]. Another pathway involved in EMT in COPD is the urokinase-type plasminogen activator (uPA)/urokinase-type plasminogen activator receptor (uPAR)-dependent cell signalling pathway. Wang Q et al. have documented an increased uPAR expression, associated with an increased EMT activity, in the small airway epithelium of COPD patientsas compared with non-smokers and smokers with normal lung function [201]. Moreover, in a subsequent study, the same authors showed that uPA is also upregulated in human small airway epithelial cell lines (HSAEpiCs), as well as in the small airways epithelium of COPD patients and is correlated with vimentin expression at this level. uPA and uPAR inhibition was able to inhibit CSE-induced EMT in HSAEpiCs [202]. These findings thus suggest that the activation of the uPA/uPAR pathway might represent a novel mechanism involved in EMT development and in airway remodelling in COPD. Interestingly, in a retrospective study, uPAR expression was also found increased in pulmonary macrophages and alveolar cells from COPD patients compared to controls, and it was also positively correlated with the levels of collagen [203]. It is important to highlight that other pathways may be involved in the induction of EMT by cigarette smoking in COPD; moreover, as we already emphasised, in addition to cigarette smoke, other environmental stresses, including the high oxidative burden and the reduced antioxidant defences, as well as the signals induced by the excessive inflammatory response or the mechanical stress can also trigger mechanisms and processes involved in EMT. Therefore, the development of EMT and the progression of fibrotic changes in the airways of COPD patients are the result of a complex network involving different triggers and multiple signalling pathways. Finally, we would like to emphasise that EMT is also active in large airways of smokers with COPD. Interestingly, Malik and colleagues documented that type III EMT is characteristic of the large airways, where RBM hypervascularisation is also observed, while type II EMT is active in the small airways, where it is involved in the fibrotic changes, contributing to the remodelling and obliteration of these airways [202]. In large airways, the type III EMT process could induce, in the context of some microenvironment alterations, the development of lung cancer that is known to be associated with COPD. Recently, a genomic link was demonstrated among COPD, lung cancer and Hedgehog signalling, which is also involved in EMT induced by tobacco-smoke [204].

**Table 2:** Animal and cells culture models suggesting EMT implication in COPD.

Models	Techniques	EMT Program	AR	EMT Signal	EMT TFs	EMT Mark	Refs.	Year
ALI HBECs (2–5 weeks) from non-smokers, smoker controls, mild, moderate and severe to very severe COPD	IHC and IF RT-qPCR WB	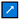 vimentin (mRNA and protein) for severe COPD and fibronectin (protein) 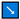 E-cadherin and ZO-1 (protein)						
	ELISA							
		+TGF-β1 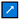 vimentin and fibronectin (protein) +blocking TGF-β1 Restoring a cobblestone shape compared with the spindle shape 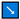 vimentin expression	x	x		x	[171]	2015
ALI HBECs from non-smokers, smokers and patients with COPD	IHC and IF RT-qPCR WB ELISA	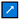 α-SMA, vimentin and collagen type I (mRNA and protein) 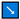 E-cadherin and ZO-1 (mRNA and protein) 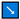 KRT5 and KRT18 (mRNA)				x	[174]	2013
ALI HBECs (14–21 days) from non-smokers, smoker controls, mild, moderate and severe to very severe COPD	RNA-seq WB ELISA IHC and IF	Activation of WNT/β-catenin pathway with 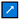 nuclear expression of β-catenin in the COPD airway epithelium 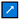 vimentin (mRNA and protein) 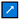 fibronectin release following WNT activation	x	x		x	[193]	2020
HBEC monolayer	RT-qPCR WB IHC and IF ELISA	+nicotine: 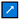 Wnt3a (protein and mRNA) 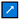 total β-catenin (protein) 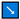 E-cadherin (protein) 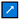 α-SMA, MMP-9 and collagen type I (protein)		x		x	[194]	2013
ALI HBECs from controls lung tissue	IF RT-qPCR WB ROS	+2.5% CSE for up to 7 days: 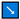 E-cadherin, ZO-1 (mRNA and protein) 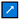 vimentin, collagen type I and α-SMA (mRNA and protein) 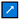 GTP-Rac1 and pAKT (protein)	x	x		x	[196]	2015
Male BALB/c mice 6 weeks	IHC WB	+CSE: collagen deposition 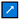 CRAMP and vimentin 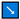 E-cadherin 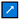 TACE, TGF-α and EGFR (protein)	x	x		x	[197]	2021
NCI-H292 cell line	IF	+CSE 5%: 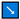 E-cadherin 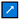 vimentin				x	[197]	2021
male C57BL/6 mice 8 weeks	IHC and IF RT-qPCR WB	20 CS/day 12 or 24 weeks: inflammation 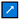 ECM, smooth muscle thickening, goblet cell hyperplasia and mucus secretion 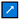 IL-17A and C-EBPβ 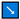 E-cadherin (mRNA and protein) 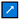 vimentin (mRNA and protein)	x			x	[200]	2021
Murine bronchial epithelial cells	IF RT-qPCR WB	20% CSE 72 h: 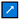 IL-17R 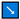 E-cadherin (mRNA and protein) 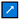 vimentin (mRNA and protein)				x	[200]	2021
HSAEpiCs	RT-qPCR WB	5% CSE 24–96 h: 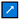 α-SMA, N-cadherin and uPAR (protein) 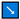 E-cadherin and α-catenin (protein)				x	[201]	2013
BEAS-2B cell line	WB RT-qPCR	+CSE 1% 24 h: 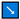 ZO-1 (protein and mRNA) 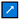 vimentin (mRNA) 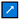 TGF-β1 (mRNA) +TGF-β1 230 pg/mL 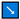 ZO-1 (mRNA)				x	[205]	2020
HSAEpiC	WB RT-qPCR	+CSE 1% 24 h: 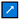 vimentin (mRNA) 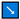 ZO-1 (mRNA) 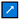 TGF-β1 (mRNA) and p-Smad2/3 (protein) +TGF-β1 230 pg/mL, 2 h 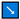 ZO-1 (protein and mRNA)		x		x	[205]	2020
Male and female Sprague Dawley rats (8 weeks)	ELISA IHC WB	+48 CS/day inhalation 12 weeks: 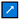 IL-8, IL-6, TNF-α, sICAM-1 and ROS in BALF Airway fibrosis, airway epithelial thickness and ASM thickness 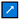 α-SMA (protein) 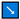 lung function 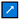 TGF-β1 and p-Smad2/3 (protein) 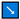 PPAR-γ (protein)	x	x		x	[206]	2021
Male C57BL/6 mice 26–28 weeks	ELISA RT-qPCR WB	+CSE intraperitoneally injected: airway epithelium thickening, enlargement of alveolus and inflammatory cell infiltration 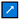 IL-6 and TNF-α in BALF 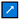 TGF-β1, Smad2 and Smad3 (protein and mRNA)	x	x			[207]	2019

α-SMA, Alpha Smooth Muscle Actin; ALI, air–liquid interface culture; AR, airway remodelling; ASM, airway smooth muscle; BALF, bronchoalveolar lavage fluid; COPD, chronic obstructive pulmonary disease; CRAMP, cathelin-related antimicrobial peptide; CS, cigarette smoke; CSE, cigarette smoke extract; EGFR: epithelial growth factor receptor; ELISA, enzyme-linked immunosorbent assay; HBECs, human bronchial epithelial cells; HSAEpiC, human small airway epithelial cells; IF, immunofluorescence staining; IHC, immunohistochemistry; IL, interleukin; KRT, keratin; Mark, EMT-markers; MMP, matrix metalloproteinase; pERK, phospho extracellular signal-regulated kinase; PPAR, Peroxisome-proliferator-activated Receptor; RT-qPCR, quantitative real-time PCR; Signals, EMT-inducing signals; TNF, tumour necrosis factor; TFs, EMT transcription factors; TGF, Transforming Growth Factor; WB, Western blot; ZO, zonula occludens. Lung tissue was obtained from patients undergoing lung resection for appropriate clinical indications.

**Table 3:** In situ evidence for EMT implication in COPD.

Models	Techniques	EMT Program	AR	EMT Signal	EMT TFs	EMT Mark	Refs.	Year
Bronchial biopsy from non-smokers, smoker controls and COPD subjects	IHC	Correlation between β-catenin and SNAIL1 expression with both S100A4 and also airflow obstruction		x	x	x	[169]	2017
Lung tissue of α1-antitrypsin-deficiency-related COPD and non- α -1 antitrypsin deficiency COPD subjects	RT-PCR	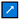 SNAIL homolog 1 in α1-antitrypsin-deficiency-related COPD group					[170]	2012
Lung tissue of non-smokers, smoker controls, mild, moderate and severe-to-very-severe COPD	IHC	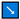 E-cadherin expression 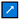 vimentin in large and small airways Negative correlation between vimentin and airway obstruction				x	[171]	2015
Lung tissue of COPD subjects	WB IHC	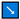 E-cadherin 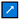 α-SMA, N-cadherin and vimentin Fragmentation and clefts in RBM	x			x	[172]	2021
Bronchial biopsy from COPD patients	IHC	Cytokeratin-(s) and S100A4 double staining				x	[177]	2011
Brushing from non-smokers, smoker controls, COPD subjects	RT-PCR IHC	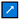 TGF-β1 (protein and mRNA) Positive correlation between TGF-β1 mRNA levels and the extent of smoking history		x			[180]	2001
Bronchial biopsy	IHC	RBM fragmentation 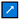 S100A4 and MMP-9 in RBM and/or basal epithelium +inhaled corticosteroids treatment: 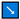 EGFR 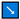 %RBM fragmentation 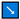 S100A4 and MMP-9 in RBM and/or basal epithelium	x			x	[185]	2014
Lung tissue of non-smokers, smokers and patients with COPD (smoker or ex-smoker)	IHC	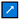 lamina propria and adventitia thickness in small airways of COPD subjects						
		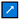 α-SMA-positive cells (myofibroblasts) in SA 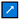 collagen-1 and fibronectin deposition Negative correlation between increased SA wall thickening and decrease in airflow in the COPD groups Correlation between collagen-1 deposition in the SA *lamina propria* and lung function in the COPD-smokers group	x			x	[186]	2021
Lung tissue of non-smokers, smoker controls, mild, moderate and severe-to-very-severe COPD	IHC and IF RT-PCR	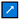 β-catenin expression in the COPD airway epithelium β-catenin upregulation in COPD airway epithelium correlates with altered differentiation	x	x			[193]	2020
Lung tissue of non-smokers, smoker controls and COPD subjects	IHC	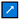 vimentin 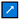 uPAR Negative correlation between FEV1% and uPAR expression Positive correlation between uPAR and the number of vimentin-positive cells				x	[201]	2013
Lung tissue of controls or subjects with chronic airflow limitation	IHC	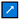 vimentin and S100A4 in SA of COPD S100A4 expression associated with airflow obstruction in small airway				x	[202]	2015
Bronchial biopsy from non-smokers, smoker controls and COPD subjects	IHC	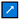 TGF-β1 in large airway Correlations between pSmad 2/3 and pSmad 7 expression and both S100A4 and airflow obstruction		x		x	[208]	2017
Lung tissue of non-COPD and patients with COPD	IHC	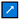 TGF-β1 (protein and mRNA)		x			[209]	1998

α-SMA, Alpha Smooth Muscle Actin; AR, airway remodelling; COPD, chronic obstructive pulmonary disease; EGFR: epithelial growth factor receptor; FEV, forced expiratory volume; IF, immunofluorescence staining; IHC, immunohistochemistry; Mark, EMT-markers; MMP, matrix metalloproteinase; NOX, nicotinamide adenine dinucleotide phosphate oxidase; uPAR, urokinase-type plasminogen activator receptor; RBM, reticular basement membrane; RT-qPCR, quantitative real-time PCR; SA, small airway; Signals, EMT-inducing signals; TFs, EMT transcription factors; TGF, Transforming Growth Factor; ZO, zonula occludens. Lung tissues were obtained from patients undergoing lung resection for appropriate clinical indications.

### 4.3. Potential Effect of Treatment on EMT and Small Airway Fibrosis in COPD

Pharmacological strategies for COPD include the use of bronchodilators and, when indicated, also ICS. Other drugs used in these patients are antibiotics when indicated, methylxanthines, mucolytics and antioxidant agents, and phosphodiesterase-4 (PDE4) inhibitors.

Despite these therapeutic approaches, it is known that COPD continue to worsen over time with the progression of lung damage and the impairment of lung function. Thus, it would be very important to develop novel therapeutic strategies addressing targets and mechanisms involved in the development of the EMT process and the fibrotic alterations in COPD airways that play a crucial role in airway remodelling and obstruction. However, few studies have investigated the effects of potential therapeutic strategies so far, either alone or in combination with other therapies, on these targets, and additional data as well as preclinical and clinical trials addressing this topic are needed.

Sohal et al. performed a proof-of-concept randomised controlled study with ICS administered for ≥6 months and showed a regression of typical EMT alterations, such as epithelial activation, RBM fragmentation and EMT biomarkers in treated patients compared to the placebo group [185].

As already discussed, it has been suggested that type III EMT may play a role in the development of cancer in COPD patients. From an analysis of nine prospective cohorts, Fan Ge et al. suggested a protective effect of ICS against lung cancer in COPD patients [210]. However, further studies on larger numbers of patients are needed to confirm these data.

Recently, Zhu et al. have shown that N-acetylcysteine, an antioxidant and mucolytic agent, was able to reduce α-SMA levels, collagen volume, wall thickness and bronchioles diameter in a COPD rat model. Furthermore, the drug was shown to inhibit the EMT process and promote immune response by acting on the VWF/p38 MAPK axis; the authors thus suggest that this drug might improve fibrotic changes in COPD [211].

Roflumilast is a PDE4 inhibitor that may be used in severe COPD associated with chronic bronchitis and frequent exacerbations. Martorana et al. demonstrated that this molecule may decrease lung damage and emphysematous changes induced by cigarette smoking in mice. They also observed a decrease in macrophage density in treated mice, suggesting that the anti-inflammatory effect of this drug might contribute to the hindering of EMT in COPD [212]. Milara et al. also showed that PDE4 inhibitors, in particular Roflumilast N-oxide (RNO), mediated a protective effect against EMT induced by cigarette smoking in bronchial epithelial cells; interestingly, this effect was also observed in primary human BECs isolated from the smokers and COPD patients’ small bronchi. Moreover, RNO induced a reduction in ROS, in NADPH Oxidase 4 expression, in TGF-β1 release, as well as in SMAD3/ERK1/2 phosphorylation induced by cigarette smoke [213]. It has also been shown that the addition of statins (simvastatin) seems to enhance in vitro the inhibitory effect of RNO against cigarette-smoking-induced EMT in human BECs [213]. Several other molecules and novel therapeutic approaches with potential inhibitory effects on the EMT process have been recently studied, such as celecoxib, a selective COX2 inhibitor [214,215]; galunisertib, a TGF-β receptor 1 inhibitor [216]; all-trans retinoic acid [217] and N-cadherin antagonist ADH-1 [218]. Most of these molecules have emerged in the oncological field over the last years and are in the preclinical or clinical phase for various solid tumours; however, in the context of their effects on inhibiting or preventing EMT induction, some of these compounds might also have a potential role in inhibiting EMT development and fibrotic changes in COPD [11].

Moreover, antifibrotic drugs approved for the treatment of idiopathic pulmonary fibrosis (pirfenidone and nintedanib) have been reported to have an inhibitory effect on EMT mainly through the TGF-β pathway [219,220].

Thus, several EMT-targeted therapies have the potential to be effective in preventing or modulating the pathological changes induced by EMT and the other processes involved in fibrotic changes in COPD. However, further preclinical and clinical studies are needed to evaluate and validate the potential benefits of these therapies in COPD.

## 5. Cystic Fibrosis and NCFB

CF is a rare genetic disease affecting over 100,000 people worldwide [221]. Mutations in the *Cystic Fibrosis Transmembrane Conductance Regulator* (CFTR) gene lead to a defect in the expression or function of the CFTR protein involved in the regulation of transepithelial ions and fluid transports. Although it is a multi-systemic disease, the main cause of morbidity and mortality in patients with CF (pwCF) is lung disease. Airway involvement with thick mucus obstructing the bronchi and an impairment of mucociliary clearance promote cycles of inflammation and infection by pathogens, such as *Staphylococcus aureus* and/or *Pseudomonas aeruginosa* [222]. The chronicity of this infectious/inflammatory process results in progressive lung damage with the development of alterations of the airway walls and a structural remodelling observed on autopsy studies or CF bronchial biopsies or lung explants and involving squamous metaplasia [223], subepithelial fibrosis [224], submucosa mucus gland enlargement [225], hyperplasia of ASM [226] and RBM thickening [227]. The order of events leading to the structural airway changes in CF and their relationship to infection and inflammation has long been debated. The observation of an early inflammatory response [228] suggests that inflammation develops prior to infection and that CFTR dysfunction is associated with a dysregulation of inflammation and is involved in the alterations of airway structure.

### 5.1. CF Inflammation

CF is characterised by the dehydration and acidification of the airway surface liquid and the hyperconcentration of mucus in the lungs. CFTR dysfunction or defect severely compromises the airway microenvironment and ultimately leads to structural lung damage and obstruction of the airways which favours persistent infection and a chronic inflammatory process [229]. The formation of mucus plugs in CF airways can trigger airway inflammation per se, even in the absence of bacterial infection and very early in the natural history of the CF lung disease. In muco-obstructive diseases [228], such as CF, a “vicious” circle occurs: mucus plugs activate lung-resident macrophages, inducing the release of IL-1β and producing a hypoxic microenvironment with necrosis of airway epithelial cells that will release IL-1α. Both IL-1α and β will activate epithelial IL-1 receptors, inducing mucin biosynthesis and the expression of pro-inflammatory cytokines and chemokines, such as IL-8, thus further amplifying the inflammatory response.

CF airway inflammation is mainly characterised by a marked and persistent recruitment of neutrophils into the airways; these cells play a crucial role in lung damage in CF. In fact, they release several noxious mediators, including proteases, such as neutrophil elastase (NE), ROS, DNA and inflammatory mediators, thus enhancing and perpetuating the chronic inflammatory process and tissue damage. NE is a serine protease actively participating in the degradation of lung tissue, and its levels have been associated with a decline of lung function [230]. It is also a predictive biomarker for the development of bronchiectasis in children [231]. The addition of NE to cultures of BECs from pwCF results in a concentration-dependent delayed/inhibited repair process, and it has been shown that alpha-1-antitrypsin, by inhibiting NE, leads to faster tissue repair [232]. Furthermore, the high levels of NE released by neutrophils induce a bacteria-killing defect by cleaving proteins (i.e., elafin and SLPI) that are involved in antimicrobial and anti-inflammatory responses [233]. A protease/antiprotease imbalance [234] occurs in CF as a consequence of the high protease burden, mainly due to the release of NE and MMPs. Furthermore, antiproteases are degraded by the proteases released by inflammatory cells and pathogens, thus further reinforcing the imbalance and the subsequent structural damage. Neutrophils also produce defensins, which activate MMPs, which normally participate in ECM degradation (collagen, elastin and gelatin) and in cytokines expression [235]. In CF, the overexpression of MMPs (MMP-8, MMP-9 and MMP-12) correlates with the impairment of lung function; in particular, MMP-9 expression has been shown to correlate with RBM degradation, the onset of bronchiectasis and a decline of lung function in pwCF [236,237,238,239]. The proteolytic activity of MMPs is also directed against CFTR [240], affecting its function and which could further aggravate the lung damage.

Some evidence suggests that the excessive and abnormal inflammatory response in CF may lead to airway remodelling [241]. In CFTR knock-out mice, chronic *P. aeruginosa* LPS exposure induced an enhanced inflammatory response that was associated with an increased susceptibility to the development of lung remodelling (increase in goblet cells and fibrosis) [242].

### 5.2. Future Directions on the Role of EMT in CF

Few papers have investigated the involvement of EMT in CF airway remodelling, while in other pathologies, a link between CFTR and signalling pathways also implicated in EMT has been demonstrated (reviewed in [12,243] and described in Table 4). In breast cancer, the downregulation of CFTR expression seems to favour cancer development and EMT induction [244,245,246,247]. Furthermore, a decreased CFTR expression leads to increased WNT signalling in mouse lung development [248]. On the contrary, CFTR knockdown in HEK293 cells was associated with a significant reduction in WNT signalling in the context of haematopoiesis [249]. So far, although the CFTR protein interacts with several actors involved in type II EMT, there is no clear evidence of its direct involvement in this mechanism [250].

Collin et al. observed an alteration in cell differentiation in the lung explants of pwCF, with an increase in mucin 5AC labelling (goblet cells) and a decrease in β-tubulin-positive cells (ciliated cells). These alterations were associated with an increase in vimentin-positive cells, suggesting EMT-related remodelling in CF lungs [251]. A transcriptome meta-analysis also revealed an EMT signature in CF epithelium and the identified protein tyrosine phosphatase *PTP*4A1/2 as being potentially involved [253].

Since the identification of the *CFTR* gene in 1989 [254,255], it has been shown that genotype alone cannot explain the phenotypic variations in CF [256]. In addition to the socio-environmental impact [257,258], the presence of modifier genes can indeed affect the pathophysiological features of pwCF and contribute to the severity of lung disease [259,260,261,262,263,264,265]. TGF-β is one of the major modifier genes and plays a critical role into myofibroblast differentiation [266]. Harris et al. showed an increase in myofibroblasts in CF lung explants that correlated with TGF-β [267]. In CF genome, three single-nucleotide polymorphisms in the *TGF-β* gene at position −509 (C or T; promoter region), +869 (T or C; codon 10 leucine or proline) and +915 (G or C; codon 15 arginine or proline) were identified [268,269,270]. The presence of proline at both sites induces low TGF-β1 expression [271], while leucine on codon 10 is associated with a high protein level [271,272,273]. TGF-β1 overexpression in pwCF has been associated with more a rapid deterioration of lung function [270]. Similarly, an increase in TGF-β1 in BALF, sputum, serum or plasma is associated with increased inflammation, some bacterial infections and the severity of pulmonary manifestations [271,274,275,276,277,278,279]. CFTR knock-out mice express more TGF-β protein in their lung tissue compared to wild-type mice [242]. Furthermore, these mice developed a more pronounced fibrotic signature with expression of ECM proteins in response to chronic LPS exposure [242]. All these observations suggest a link between CFTR dysfunction and TGF-β upregulation. RBM thickness has been shown to be positively correlated with the levels of TGF-β1 in BALF in children with CF [227].

In addition to TGF-β, other TGF-β-related players are deregulated in CF. Recently tissue transglutaminase (TG2) was identified in vitro as a regulator of TGF-β1 [252]. TG2 inhibition leads indeed to a significant reduction in fibronectin, N-cadherin, SNAIL and TGF-β1 expression, resulting in a reversal of EMT. In human BEC line (16HBE14o-), miR1343 directly represses the activity of both TGFBR1 and TGFBR2 receptors by binding to the 3’UTR region and leading to the inhibition of canonical TGF-β signalling pathways [280].

FAM13A has been suggested to be a modifier gene for CF lung phenotype; the expression of FAM13A is downregulated in CF human BEC, and this decrease is associated with a decrease in E-cadherin [281], suggesting that FAM13A could be involved in EMT modulation in CF epithelial cells.

Recently, Quaresma et al. studied the presence of EMT features in CF tissue, primary cultures of human BEC and cells lines expressing mutant CFTR [250]. In CF tissue/cells defective of CFTR, mesenchymal markers, such as vimentin and N-cadherin, were upregulated, but most epithelial markers were not repressed, suggesting that partial EMT was active. Other features of active EMT were observed, including impaired wound healing, destructured epithelial proteins and defective cell junctions, as well as the upregulation of EMT-associated transcription factors. This study also shows that the observed EMT features were mediated by the EMT-associated transcription factor TWIST1. Interestingly, mutant CFTR has also been associated with increased WNT/β-catenin signalling and an exaggerated TGF-β secretion. Most recently, the same authors identified the Hippo-associated protein Yes-associated protein 1 as a potential driver of EMT and fibrosis in CF by using a multi-omics systems biology approach [282]. This transcription factor appears to be upregulated in CF as opposed to non-CF cells, and it has been shown to impair F508del CFTR trafficking, through the interaction with this protein. Furthermore, in the same study, five potential pathways were suggested to be involved in the link between mutant CFTR and EMT; these pathways (the Hippo, WNT, TGF-β, p53 pathways and MYC signalling) would need to be further investigated as potential therapeutic targets of EMT in CF.

Sousa et al. studied the protein Kruppel-like factors (KLF4), which is a transcription factor involved in the regulation of the proliferation, differentiation and wound-healing processes that are altered in CF [283]. The depletion of KLF4 in wild-type or F508del BEC had a different impact on epithelial integrity: in wild-type KLF4 KO cells, a decrease in transepithelial electrical resistance and no effect on wound closure were observed, while in F508del-KLF4 KO cells, higher levels of transepithelial electrical resistance were observed associated with a decrease in wound closure. Furthermore, the expression of EMT biomarkers and EMT-associated TFs were also differently affected. KLF4 depletion induced a switch of epithelial protein expression with a decrease in E-cadherin and cytokeratin 18 (epithelial markers) and an increase in N-cadherin and vimentin (mesenchymal proteins), that was associated with a marked decrease in TWIST1 in F508del-CFTR cells, but not in wild-type cells.

All these observations suggest that the pathways involved in the EMT process in CF are multiple and complex, and need further investigations.

### 5.3. Potential Effect of Novel Pharmacological Treatments on EMT and CF Airway Remodelling

Until the introduction of CFTR modulators in 2012 in the USA, the pharmacological treatment of CF lung disease was symptomatic and based on anti-inflammatory drugs, antibiotics and mucolytics/airway surface liquid hydrators. These treatments have significantly improved the life expectancy and quality of life of pwCF; however, the recent introduction of CFTR modulators had an impressive impact on clinical outcomes and the lives of pwCF [284]. This novel pharmacological approach aims at correcting the basic CFTR defect, in particular at correcting the expression (correctors) and/or improving the function (potentiators) of the CFTR protein in the respiratory epithelium. Several combinations of modulators have been developed. Among these, the most recently introduced in clinical practice was Elexacaftor–Tezacaftor–Ivacaftor (ETI), which led to an impressive improvement in CF clinical outcomes.

Some recent studies suggest a potential effect of CFTR modulators on CF airway remodelling, although the data are still scarce and need to be confirmed. Adam et al., in an in vitro study, have shown that Lumacaftor–Ivacaftor (the first CFTR modulators association commercialised) accelerated airway epithelial wound repair and improved transepithelial electrical resistance in the absence and even in the presence of *Pseudomonas aeruginosa* exoproducts [285]. Interestingly, more recently, a similar effect was reported in vitro in a CF cell line overexpressing CFTR-F508del treated with ETI. In fact, this drug not only restored F508del CFTR maturation and function but also enhanced wound repair in these cells [286]. These findings seem to indicate the potential of CFTR modulators in enhancing reparative and regenerative processes into the airways. Bec et al. studied adult pwCF treated with ETI for one year and documented that the overall CT score decreased as a consequence of decreased mucus plugging and peribronchial thickening, while bronchial, parenchymal, and hyperinflation scores remained unchanged [287]. Although some few and indirect evidence concerning a potential effect of modulators on mechanisms and features of airways remodelling in CF seem encouraging, further studies need to address this topic; longitudinal studies with CFTR modulators could provide further insight into their effects as well as on mechanisms involved in the EMT process associated with CF.

### 5.4. Inflammation and EMT in NCFB

NCFB are muco-obstructive diseases, including a heterogenous group of diseases with a number of underlying conditions, that affect patients of different age. The prevalence of the disease is increasing worldwide [288,289]; however, in 38.1% of cases of NCFB, the aetiology is not yet identified [290]. Similarly to CF lung disease, pathophysiology of bronchiectasis is linked to a vicious vortex of impaired mucociliary clearance, airway inflammation and infection, ultimately leading to structural damage. In particular, an aberrant epithelial remodelling with impaired mucociliary escalator architecture is present in both large and small airways. As in CF, in NCFB patients, their mucus is dehydrated and more viscous, favouring the development of infections. A marked neutrophilic inflammation is observed in the airways of patients with (pw)NCFB, and similarly to CF, a protease/antiprotease imbalance occurs with the release of NE and the overexpression of MMPs that correlate with impaired lung function and the increased neutrophilic inflammation [291]. Furthermore, severe disease was shown to be associated with an upregulation of neutrophils and NET [292].

Eosinophilic inflammation has also been observed in approximately 20% of NCFB patients and may be involved in disease exacerbations. Moreover, in pwNCFB polymorphism at position −1607 (1G or 2G) of the MMP-1 promoter induces the upregulation of MMP-1 activity, which is associated with post-inflammatory lung destruction and fibrogenesis. In a small cohort of pwNCFB, it has been shown that 1G/1G or 1G/2G genotypes are associated with a more severe phenotype and higher serum levels of MMP-1 and TGF-β1 [293].

Although airway remodelling and structural damage are key components of NCFB, so far, there are no data relating these features to the presence of EMT in these diseases, but this is certainly a field that needs active investigations as it could potentially suggest novel treatment approaches.

## 6. Conclusions

Chronic obstructive airway diseases are characterised by an exuberant and persistent inflammatory process into the airways as the result of repeated aggression by different triggers and noxious agents, such as cigarette smoking, pollutants, allergens and pathogens. In the context of this chronic inflammatory environment, tissue repair becomes excessive and altered, leading to the development of structural alterations, such as remodelling and fibrotic changes with different degrees of reversibility over time; these changes ultimately lead to organ dysfunction and the progressive impairment of lung function.

Although the role of EMT in the pathogenesis of these diseases is not yet well defined, several studies seem to suggest that this process is involved in pathological changes and airway remodelling, leading to chronic airflow obstruction and progressive lung function impairment.

Even if common signalling pathways are implicated in structural changes observed in these diseases, some specific triggers and a different regulation may be operating. Thus, it would be important to better define, according to the guidelines for research on EMT [1], the different mechanisms and pathways involved in the EMT process in these different airway diseases in order to identify potential specific therapeutic targets to prevent or limit the remodelling process and the irreversible structural changes.

## Figures and Tables

**Table 4:** Potential indicators of EMT implication in CF models and CF lung explants.

Models	Techniques	EMT Program	AR	EMT Signal	EMT TFs	EMT Mark	Refs.	Year
Primary HBECs (from explanted CF lung or from control subjects who underwent lung surgery)	TEER IF RT-qPCR WB Wound healing	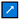 N-cadherin and vimentin (protein) 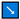 TEER	**x**			**x**	[250]	2020
CFBE41o-wt or -F508del-CFTR HEK 293T cells	IF WB Wound healing	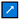 N-cadherin vimentin and collagen I (protein) 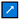 ZO-1 and CX31, while 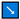 claudin-1 and Desmoplakin I/II Multilayered organisation for CF versus monolayer for control EMT markers localisation differences 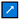 Ki-67-positive cells in basal cell layers 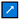 time to close the wounds 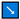 TEER 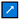 TWIST1 (protein) +CFTR modulator treatments 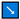 N-cadherin and vimentin 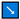 TWIST1 +response to TGF-β1: No difference in response 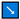 CK18 and 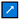 N-cadherin (protein) WT more resistant to EMT induction than CF TWIST1 shRNA knockdown in HEK293T cells: vimentin inhibition	**x**	**x**		**x**	[250]	2020
CF versus control lung tissues	IF RT-qPCR	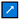 occludin, tight junction protein 1/zonula occludens-1, connexin 43, connexin 26 and cytokeratin 18 (mRNA) 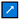 vimentin (mRNA) Polygonal flat cells on the CF epithelial layer surface						
		Fewer cylindrical-shaped columnar cells and several cell layers in CF tissue 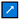 TWIST1 and ZEB1 (mRNA) Positive staining for SNAILl + Slug and ZEB1 Partial EMT	**x**			**x**	[250]	2020
CF versus control lung tissue	IHC	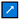 MUC5AC (protein) 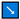 β-tubulin (protein) 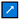 vimentin-positive spindle-shaped Thickening of the RBM	**x**			**x**	[251]	2021
ALI HBECs (CF and controls lung tissue 2 weeks)	TEER WB ELISA RT-qPCR IHC	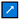 MUC5AC (protein) 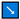 MCIDAS, MYB and FOXJ1 (mRNA) 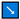 TEER 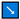 E-cadherin and occludin Spindle-shaped cells and thickness of epithelium +CFTR inhibition or ***Pseudomonas*** infection in control culture No EMT induction	**x**			**x**		
IB3-1 cells F508del/W1282X C38 cells as control	Cell migration assay WB IHC RT-qPCR ELISA	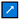 fibronectin, N-cadherin and the transcription repressor Slug in IB3 cells compared to the C38 cells 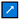 migratory phenotype 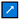 TGF-β1 (mRNA and protein) +TGF-β receptor inhibitor 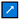 fibronectin (protein) +TG2 inhibitors in IB3-1 cells Inhibition of cell migration 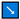 TGF-β1 (protein)	**x**	**x**	**x**	**x**	[252]	2016
ALI HBECs 14 days	WB	+TGF-β1 3 ng/mL 48 h: 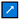 TG2 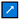 Fibronectin and N-cadherin 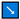 E-cadherin (protein) +TG2 overexpression: 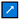 fibronectin, N-cadherin, Slug (protein) 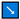 E-cadherin (protein) 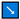 TEER				**x**	[252]	2016

AR, airway remodelling; CF, cystic fibrosis; CFBE, CF Bronchial Epithelial Cell Line; CFTR, cystic fibrosis transmembrane conductance regulator; EMT, epithelial–mesenchymal transition; FOXJ, Forkhead box J protein; HEK, human embryonic kidney; IF, immunofluorescence staining; IHC, immunohistochemistry; Mark, EMT-markers; MCIDAS, Multiciliate Differentiation And DNA Synthesis-associated Cell Cycle Protein; MUC, mucin; RBM, reticular basement membrane: RT-qPCR, quantitative real-time PCR; Signal, EMT-inducing signals; TEER, transepithelial electrical resistance; TFs, EMT transcription factors; TG, transglutaminase; TGF, Transforming Growth Factor; ZEB, Zinc finger E-box binding homeobox; ZO, Zonula occludens. Lung tissues were obtained from subjects undergoing lung resection for appropriate clinical indications or from CF patients at transplantation.

## Data Availability

Not applicable.
